# The physiological functions of central nervous system pericytes and a potential role in pain

**DOI:** 10.12688/f1000research.13548.1

**Published:** 2018-03-20

**Authors:** Nicholas Beazley-Long, Alexandra M Durrant, Matthew N Swift, Lucy F Donaldson

**Affiliations:** 1 Arthritis Research UK Pain Centre & School of Life Sciences, Medical School, University of Nottingham, Nottingham, UK

**Keywords:** Pericytes, Central Nervous System, Pain

## Abstract

Central nervous system (CNS) pericytes regulate critical functions of the neurovascular unit in health and disease. CNS pericytes are an attractive pharmacological target for their position within the neurovasculature and for their role in neuroinflammation. Whether the function of CNS pericytes also affects pain states and nociceptive mechanisms is currently not understood. Could it be that pericytes hold the key to pain associated with CNS blood vessel dysfunction? This article reviews recent findings on the important physiological functions of CNS pericytes and highlights how these neurovascular functions could be linked to pain states.

## Introduction

Other than the retina, the central nervous system (CNS) contains the highest ratio of pericytes to endothelial cells in the body ^1^, yet the contribution, if any, of CNS pericytes to pain states and nociception is not understood. CNS pericytes inhabit a perivascular niche within the neurovascular unit (NVU) ^2^, a unique position interfacing the circulatory and peripheral immune systems and the central nervous parenchyma. CNS pericytes regulate critical functions of the NVU: blood-brain barrier/blood-spinal cord barrier (BBB/BSCB) integrity, cerebral (and presumably spinal cord) blood flow, clearance of toxic substances, angiogenesis, mesenchymal stem cell activity, and neuroinflammation ^3^. As such, severe neuronal defects are observed with CNS pericyte deficiency ^4, 5^. CNS pericytes have attracted interest in neuropharmacology, particularly with respect to their involvement in neuroinflammation, yet on the basis of a scan of the published literature on CNS pericytes, it is evident that understanding of their potential influence(s) in pain states and nociceptive mechanisms is currently severely lacking. This article reviews recent literature on the physiological functions of CNS pericytes that, when awry, could contribute or lead to the development of pain.

## The multipotent nature of CNS pericytes

Pericytes, first identified and labelled as Rouget cells in 1873 by the French physiologist Charles-Marie Benjamin Rouget, are a heterogeneous population of cells and, as such, have proven a challenge to characterise both functionally and biochemically. A lack of pericyte-specific markers has significantly hindered consistency within pericyte research, and many debates discussing what constitutes a pericyte have played out ^6^. Pericytes are capable of self-renewal, and express markers and behave like mesenchymal stem cells. For example, throughout the body, pericytes have the ability to replace specialized tissue-specific cells such as adipocytes ^7^, myocytes ^8^, myofibroblasts ^9^ and odontoblasts ^10^ in repair processes. Pericytes can also facilitate repair processes indirectly through the release of factors ^11, 12^, and some of these – for example, nerve growth factor (NGF), vascular endothelial growth factor-A (VEGF-A), tumour necrosis factor-alpha (TNFα), interleukin 1 beta (IL1β), IL6, NAD(P)H oxidase-4 (NOX4) and matrix metalloproteinase 2 (MMP2) ^13– 18^ – are direct neuronal sensitizers or increased levels are associated with pain states. CNS pericytes have been shown to migrate into the cortex parenchyma and differentiate into a microglia-like phenotype in a model of stroke ^19^. The authors observed pericyte migration, proliferation, a morphological change resembling reactive microglia, and expression of IBA-1 and CD11b, the latter being an integrin strongly expressed by reactive microglia and macrophages in pain models ^20– 22^. However, it is not known whether such pericyte-to-microglia differentiation occurs in pain states in which microglial activation and central sensitization occur. Microglial blockage (with minocycline, for example) can exhibit anti-nociceptive actions in pain models ^23^. A pericytic transformation into a pro-nociceptive microglial phenotype would present a novel mechanism to target for alleviation of microglial-driven neuroinflammation and neuronal sensitization known to underpin some chronic pain states, in preclinical models and in humans ^24– 27^.

To complicate matters further, CNS pericytes can differentiate into a neuronal-like phenotype with basic fibroblast growth factor (bFGF) stimulation and are also capable of self-renewal ^28^, indicating that pericytes may be a source of pluripotent progenitor cells. Forebrain pericytes are of neuroectodermal origin ^29^ and it may be this pericyte subtype that gives rise to neuronal phenotypes. The heterogeneous and pluripotent nature of pericytes appears to allow diverse differentiation responses in different situations. How CNS pericytes behave in pain states and preclinical pain models and whether they present a novel target for the alleviation of pain are not yet known.

## CNS pericytes in vessel barrier integrity

**Figure 1.**
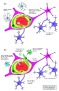
A diagrammatic overview of the physiological roles of central nervous system (CNS) pericytes and possible links of pericyte function to neuronal sensitization and pain. ( **A**) Under physiological conditions, the high pericyte-vessel coverage in the CNS promotes high tight junction protein expression, consequently maintaining vessel integrity and reduced vessel permeability. Pericytes influence the low level of blood cell transmigration into the parenchyma under physiological conditions. ( **B**) Reduced pericyte coverage in many CNS diseases leads to decreased tight junction protein expression, loss of vessel integrity, and increased vessel permeability. Ensuing pro-nociceptive molecule extravasation and pro-nociceptive and pro-inflammatory immune cell transmigration are likely to lead to neuronal sensitization. In addition, there is emerging evidence that multipotent CNS pericytes are able to migrate out of their peri-vascular niche and differentiate into a microglia-like phenotype in preclinical pain models, which in turn could have a neuronal sensitizing effect. A, astrocyte; BM, basement membrane; EC, endothelial cell; L, leukocyte; M, microglia; N, neuron; P, pericyte.

The BBB and BSCB are selective barriers that limit cell and molecular access into the CNS from the blood. The barriers maintain the microenvironment within the CNS required for physiological neuronal function. The CNS microvasculature is comprised of endothelial cells, pericytes, perivascular macrophages, microglia, and astrocytic end-feet ( Figure 1). Unlike in the periphery, CNS endothelial cells are not fenestrated but are connected via tight junction proteins such as occludin, junctional adhesion molecules (JAMs), vascular endothelial cadherin (VE-cadherin), and claudins, which restrict the inter-endothelial space. Pericytes are embedded in the basement membrane (perivascular niche), which surrounds the endothelial cells. Pericytes are polymorphs with an oval to elongated morphology and extend processes along capillaries, pre-capillary arterioles and post-capillary venules. In the CNS, these processes encircle the endothelium and cover endothelial tight junction regions ^30^. Astrocytic end-feet wrap around the basement membrane encircling perivascular cells and vessels and provide another barrier (glia limitans), further limiting access to the nervous parenchyma.

Pericytes are key modulators of the BBB/BSCB and participate in neuroinflammation ^3, 31^. Platelet-derived growth factor receptor-beta (PDGFRβ) is predominantly expressed by pericytes in the CNS ^32^ and, via mice with genetically disrupted PDGFRβ signalling, demonstrated the necessity for pericytes in BBB formation during embryogenesis ^33^. In addition, in both development and adulthood, barrier permeability is inversely correlated with pericyte coverage ^5, 33^. There is lower pericyte capillary coverage in the spinal cord compared with the brain, which correlates with increased permeability, and lower expression of two tight junction proteins: ZO-1 and occludin ^34^.

Mice with deficient PDGFRβ signalling ( *pdgfrβ ^F7/F7^*) demonstrated region-dependent losses in pericytes that related to BBB breakdown ^35^. Conversely, disrupted PDGFRβ signalling through a mutation in the retention motif of PDGF-B ( *pdgfb* ^ret/ret^), one of two ligands for the receptor, caused homogenous pericyte loss across the brain, but the extent of pericyte loss in this experiment did not correlate with increased BBB permeability ^36^. The authors hypothesise that this may be due to the phenotypic diversity of pericytes and alternative local signalling mechanisms controlling BBB permeability. In addition, the difference in mutation strategy (receptor versus ligand) could have contributed to the contrasting results.

Foxf2, a transcription factor, is specifically expressed in cerebral pericytes derived from the neural crest (neuroectodermal cells) ^37^. Loss of Foxf2 caused cerebral haemorrhage, increased pericyte densities in embryonic cerebral capillaries, and induced BBB disruption in both development and adulthood. There was also a decrease in PDGFRβ and transforming growth factor beta (TGFβ) (implicated in pericyte and endothelial proliferation, migration and differentiation) signalling despite an increased number of pericytes ^37^. This suggests that the correct differentiation of ** pericytes is key to BBB development and maintenance, and there are cues other than PDGFRβ which are involved in pericyte recruitment to the endothelium. For example, loss of glial laminin resulted in BBB breakdown, concluded to be due to the observed altered pericytic differentiation into a contractile phenotype, consequently disrupting the barrier ^38^. In addition, CD146 has been implicated in regulating PDGFRβ/PDGF-B and TGFβ signalling in barrier formation and maintenance. Pericyte-secreted CD146 acts as a co-receptor for PDGFRβ during pericyte-vascular recruitment, and in the mature barrier, endothelial cell–secreted CD146 is downregulated by pericyte production of TGFβ ^39^.

Pericyte-endothelial cell signalling is paramount in the maintenance of the BBB/BSCB, especially through PDGFRβ/PDGF-B signalling ^40^. However, many of the specific mechanisms of how pericyte-endothelial cell signalling affects barrier function are still largely unknown. *In vitro* culture techniques offer the ability to study pericyte function in detail. Indeed, much of the knowledge gained about pericytes has been from combined *in vivo* and *in vitro* techniques. Recently, Herland *et al*. ^41^ developed a dynamic flow model within a microfluidic device that permits co-culturing human endothelial cells in an engineered lumen with pericytes or astrocytes embedded in the surrounding extracellular matrix. In this model, the presence of pericytes reduced the permeability of the engineered vessel and increased the production of both basal and TNFα-induced cytokines compared with endothelial cells alone. The development of sophisticated *in vitro* models of the BBB/BSCB will allow more detailed and specific research into the contribution of pericytes and other cell types to barrier permeability and function.

In many preclinical models of painful neuropathy, the BBB/BSCB is altered ^42– 46^. Leakage of neurotoxic blood-derived molecules into the nervous parenchyma (for example, erythrocytic free iron, fibrinogen, plasminogen and thrombin) can lead to a detrimental neuronal response, including sensitization, and may contribute to an increased pain state in various painful diseases ( Figure 1b). Gaining a better understanding of pericytic function (or indeed pericytic dysfunction) in the loss of barrier integrity in the context of pain may present an opportunity to intervene and limit the possibly painful consequences.

## Pericytes in haemodynamic regulation

The precise roles of contractile pericytes, despite their isolation and identification in the 1870s, in regulating haemodynamic control of CNS blood flow are only now being probed effectively. Smooth muscle cell (SMC) contraction in pial and penetrating arterioles is, as in other tissues, the primary control on CNS blood flow ^47^. Capillaries are devoid of SMC and evidence indicates that pericytes contribute to blood flow regulation in capillaries, most likely through electrical coupling with capillary endothelial cells ^48, 49^. Pericytes are able to regulate bi-directional control of CNS capillary diameter independent of arterioles ^50^, and pericyte stimulation propagates signals that cause downstream pericytes to constrict, indicative of a pericyte-pericyte signalling network ^51^. Furthermore, there is evidence of an electrical endothelial network: CNS capillary endothelial cells expressing the potassium channel K _Ir_2.1 caused vasodilatation of distant upstream arterioles in the CNS microvasculature in the absence of pericytes ^52^. The authors conclude that a hyperpolarising signal is transmitted through endothelial gap junctions, inhibiting calcium influx, and causes SMC relaxation and vessel dilation. Evidence points towards pericytes being electrically coupled to capillary endothelial cells and therefore possibly being able to regulate this novel electrical endothelial network ^47, 48^. Further evidence of the intricate relationship between pericytes and capillaries being responsible for control of cerebral blood flow (CBF) following neuronal innervation derives from knockout animals, in which decreased pericyte numbers resulted in a reduction in capillary coverage and dysregulation of the microvasculature ^35, 53, 54^. Potential signalling networks between pericytes and myocytes in uterine smooth muscle also point to multi-cellular interactions in blood flow control, as pericyte constrictions persist longer following stimulation compared with myocytes ^55^. Exaggerated pericyte constriction, persisting longer than SMC constriction, has been linked to a loss of reperfusion in ischaemia and stroke, even when occluded arteries have been dilated ^56– 60^. This supports the role of pericytes having an influence on CBF which can be detrimental.

In keeping with a pericyte contribution to the NVU ^47^, several neuro-glial transmitters modulate pericyte influence on microvasculature in cerebellar slices. Pericyte populations are heterogenous depending on pericyte locus in the microcirculation ^6, 40, 53, 61, 62^. Pericyte constriction is stimulated by noradrenaline and blocked by glutamate, transmitters involved in neurovascular coupling. HETE-20 is a known CNS vasoconstrictor that is inhibited by glutamate-driven nitric oxide (NO) release. Block of synthesis of both HETE-20 and NO resulted in pericyte dilation, mediated by prostaglandin E _2_, a known CNS vasodilator ^50^.

Although the exact contribution of pericytes in maintaining and altering CBF requires further elucidation, evidence suggests that they have a much more significant role in CBF than assumed since their initial discovery. Emerging evidence points to pericytes acting as major players in the NVU which involved a “sensory web” of microvasculature ^52^. Pericytes preside over profound changes in capillary tone and may be able to initiate upstream effects on arteriolar smooth muscle, contrary to initial opinion. These findings implicate pericytes as key players in pain that arises from altered CBF, for example in migraine and chronic pain conditions associated with altered blood vessel function ^63^. Blood oxygen level-dependent technology has linked generalised cerebral hypoperfusion with severe pain in migraineurs, which was associated with concurrent vasospasm ^64^. Induced hypoxia worsened pain in response to stimuli designed to invoke an episode. Such stimuli could be linked to aberrant neuronal signalling causing detrimental pericytic action ^65^. These intriguing studies highlight how aberrant neurovascular coupling and detrimental pericytic function could contribute to pain.

## Pericytes in CNS angiogenesis

Pericytes are vital for vascular function, including the control of angiogenesis. Angiogenesis is important in the development and maintenance of CNS function and involves several cell types in the NVU. Developmental CNS angiogenesis is initially dependent on neural VEGF-A expression leading to the formation of endothelial-derived tip cells and enhanced endothelial cell survival. Pericytes form part of the NVU ^66^. They are recruited to sprouting vessels through endothelial secretion of PDGF-β, promote tube formation, and later secrete angiostatic substances that contribute to the termination of CNS angiogenesis and vascular stabilisation ^67^. The reduced permeability of the BBB compared with the systemic vasculature is not intrinsic to endothelial cells; the presence of neuronal precursors is required for BBB induction, and CNS pericytes and astrocytes are required for BBB maturation ^66^.

The contribution of pericytes to BSCB and angiogenesis is less well understood, but evidence suggests that it is important as activated pericytes stimulate increased vascular density (interpreted as angiogenesis) in spinal cord explant cultures ^68^. Altered BSCB function is evident both in people with amylotrophic lateral sclerosis (ALS) and in animal models of ALS ^69^. Patients with ALS have increased spinal cord ventral horn microvascular density (also interpreted as angiogenesis) with decreased spinal cord pericyte coverage; those patients requiring respiratory support displayed an increased incidence of spinal cord angiogenesis ^27^. These human observations imply that spinal cord vascular dysfunction, with increased angiogenesis and decreased pericyte function, contribute to the disease.

## Do pericytes contribute to migration of immune cells into the CNS and the generation of pain?

The BBB/BSCB normally restricts leukocyte entry and as a result the CNS is considered an immune-privileged site under normal conditions. However, under many pathological conditions, leukocyte transendothelial migration into the CNS occurs and pericytes contribute to this process. First, pericyte dilatation increases blood flow to specific areas, thereby increasing leukocyte delivery to the NVUs in question. Second, pericytes are able to release factors into the circulation which promote leukocyte chemoattraction, including TNFα, interleukins (including IL-1β, IL-6 and IL-10), interferon gamma (IFNγ), TGFβ1, and members of the CC (denoting 2 adjacent cysteines) chemokines, including monocyte-chemoattractant protein-1 (MCP-1) ^70, 71^. Pro-inflammatory secreted factors are, however, species-dependent, and rodents differ significantly from human pericytes in their secretome ^3^. Third, CNS pericyte-derived chemokines stimulate leukocyte integrins, allowing interaction with endothelial adhesion molecules in the vascular lumen, and pericytes also express intercellular adhesion molecule-1 (ICAM-1) and vascular cell adhesion molecule-1 (VCAM-1) contributing to leukocyte transmigration into the perivascular space ^3^. Lastly, once leukocytes are in the perivascular niche, without pericyte-mediated adhesion molecule guidance, leukocytes can be cleared by a perivascular clearance mechanism and not breach the astrocytic end-feet (glia limitans) and reach the nervous parenchyma ^2^.

In preclinical models of painful neuropathy such as peripheral nerve injury model, there is evidence that immune cells transmigrate into the CNS and these may contribute to the development of CNS neuronal sensitization (central sensitization) ^72– 74^. A recent report shows that peripheral nerve injury results in disruption of the BSCB, and loss of both tight junction proteins and spinal pericyte coverage ^75^. Therefore, if pericytes regulate the passage of immune cells into the nervous tissue parenchyma ( Figure 1), then altering this process may be a viable intervention with the aim of lessening central sensitizing processes that lead to increased pain. Pericytes are crucial to the development of the CNS and in central neurodegenerative disorders, and these findings suggest that they also contribute to spinal processing of sensory information and pain.

## Summary

This article highlights the key areas of CNS pericyte physiology that, when dysregulated in pathology, could lead to neuronal sensitization and an increased pain state ( Figure 1b). Pericytes are a more attractive pharmacological target than those that are beyond the second barrier of the BBB/BSCB, the glia limitans. An agent targeting CNS perivascular cells will not need to penetrate the glia limitans thereby reducing possible off target and detrimental side effects within the CNS parenchyma. However, whether CNS pericytic actions affect pain is currently severely under-researched; more focussed research into the actions of pericytes in the context of neuronal sensitization and pain could present many potential therapeutic opportunities.
